# Digital Health Literacy Related to COVID-19: Validation and Implementation of a Questionnaire in Hispanic University Students

**DOI:** 10.3390/ijerph19074092

**Published:** 2022-03-30

**Authors:** María F. Rivadeneira, María J. Miranda-Velasco, Hiram V. Arroyo, José D. Caicedo-Gallardo, Carmen Salvador-Pinos

**Affiliations:** 1 Institute of Public Health, Faculty of Medicine, Pontificia Universidad Católica del Ecuador, Quito 17-01-2184, Ecuador; jcaicedo707@puce.edu.ec; 2 Department of Education Sciences, Faculty of Teacher Training, University of Extremadura, 06006 Bajadoz, Spain; mirandav@unex.es; 3 School of Public Health, University of Puerto Rico, San Juan 365067, Puerto Rico; hiram.arroyo1@upr.edu; 4 School of Economics, Pontificia Universidad Católica del Ecuador, Quito 17-01-2184, Ecuador; 5 Universidad Central del Ecuador, Quito 6120, Ecuador; pochasalvador@yahoo.com

**Keywords:** digital health literacy, COVID-19, validation, questionnaire, university students

## Abstract

Digital health literacy influences decision-making in health. There are no validated instruments to evaluate the digital literacy about COVID-19 in Spanish-speaking countries. This study aimed to validate the Digital Health Literacy Instrument (DHLI) about COVID-19 adapted to Spanish (COVID-DHLI-Spanish) in university students and to describe its most important results. A cross-sectional study was developed with 2318 university students from Spain, Puerto Rico, and Ecuador. Internal consistency was measured with Cronbach’s alpha and principal component analysis. Construct validity was analyzed using Spearman’s correlations and the Kruskal–Wallis test. The internal consistency of the questionnaire was good for the global scale (Cronbach’s alpha 0.69, 95% CI 0.67) as well as for its dimensions. A total of 51.1% (*n* = 946) of students had sufficient digital literacy, 40.1% (*n* = 742) had problematic digital literacy, and 8.8% (*n* = 162) had inadequate digital literacy. The DHLI was directly and significantly correlated with age, subjective social perception, sense of coherence, and well-being (*p* < 0.001). The average digital literacy was higher in men than in women, in students older than 22 years, and in those with greater satisfaction with online information (*p* < 0.001). The COVID-DHLI-Spanish is useful for measuring the digital literacy about COVID-19 in Spanish-speaking countries. This study suggests gaps by gender and socioeconomic perception.

## 1. Introduction

The current COVID-19 pandemic has provoked an avalanche of digital information, to the point of being considered an infodemic by the World Health Organization. The large amount of information available can be overwhelming for citizens and even counterproductive if false, incomplete, or inadequate information is accepted as real. The citizen who receives the information must have sufficient elements to interpret it in its context, discriminate it, and use it for better health performance, in order to limit the spread of the disease and promote a culture of care [1].

The World Health Organization defines health literacy as “the personal characteristics and social resources necessary for individuals and communities to access, understand, evaluate, and use information and services to make decisions about health” [2]. In this sense, digital health literacy promotes the empowerment of people, based on access to adequate and timely information for health decision-making that favors their self-care and the care of others in their environment. Digital literacy also promotes equity in health, well-being, and resilience, fundamental aspects during the current pandemic by COVID-19 [2]. 

On the other hand, low digital health literacy has been associated with higher mortality [3], hospitalizations [4], lower use of and access to health services [5] as well as lower ability to make health-related decisions [6]. The health literacy has been shown to be a better predictor of health outcomes than sociodemographic determinants [7]. In the current context of the COVID-19 pandemic, empowering citizens through adequate health information is critical to take public health actions that can limit the spread of the disease and save lives.

During the pandemic, the use of digital social media such as Facebook, YouTube, and Twitter, among others, became the main source of information search [8]; worldwide, the use of such networks increased by 20–87% [8,9]. Adolescents and young adults are frequent users of these types of digital media. Previous studies have found that young adults under 30 years of age showed a greater increase in the use of these digital media after the COVID-19 pandemic compared to people 30 years of age and older [10,11]. On the other hand, young adults have been related to greater problems in complying with measures such as restricting mobility, social distancing, and the use of biosecurity measures [12]. In this sense, digital health literacy is fundamental in this population group, as it would be associated with behaviors, attitudes, and healthy decision-making in the context of the pandemic [13]. Indeed, better digital literacy has been related to compliance with preventive measures as well as a greater willingness to accept the COVID-19 vaccine [13,14,15].

According to other authors, the digital literacy on COVID-19 in young adults has important limitations, despite their constant use of digital media [16,17]. Likewise, it has been found that this population presents difficulties in judging whether the information found is reliable [16]. In America, it has been estimated that one in three adults has low digital health literacy [8]; similarly, in Europe and Asia, low levels of digital literacy have been reported, ranging from 40–49% in Europe [18,19] to 26–93% in Asian countries [20]. On the other hand, it is known that the digital literacy on health is related to sociodemographic factors, with differences found by sex, educational, and socioeconomic level [21,22], and is associated with anxiety about the future and well-being [23].

In order to better manage the ability to use information, discriminate it, and take advantage of it for the benefit of health, it is necessary to strengthen digital health literacy. One of the first steps for this is to have valid instruments that will allow us to evaluate the level of digital health literacy related to COVID-19 [8]. From these instruments, it would be possible to monitor the state of literacy related to COVID-19, and subsequently evaluate the public health strategies focused on strengthening health literacy in citizens. 

In recent years, several instruments have been developed to measure digital health literacy [24]. One of the most widely used has been the eHealth Literacy Scale, which focuses on measuring the search for and evaluation of online information [25,26]. Subsequently, van der Vaart and Drossaert proposed the Digital Health Literacy Instrument (DHLI), which evaluates six dimensions: information searching, adding self-generated content, evaluating reliability, determining relevance, and protecting privacy [27]. Based on this questionnaire, Dadackinsky and colleagues proposed the Digital Literacy Instrument (DHLI) in relation to COVID-19 [1,28,29]. The original version in English has been adapted and validated in several languages including German, [16], Portuguese [30], and Korean [31], among others, demonstrating good validity and reliability to evaluate the digital health literacy with regard to COVID-19. 

To our knowledge, there have been no previous studies evaluating the validity of a digital literacy scale in a Spanish-speaking population. The purpose of this research was to evaluate the validity of the DHLI scale for digital literacy related to COVID-19 adapted to Spanish and to analyze the main characteristics of the digital literacy around COVID-19 in university students. The novelty of this study is to have a digital health literacy measurement instrument adapted and validated for Spanish. From this instrument, it will be possible to carry out research that evaluates the digital literacy on COVID-19 in the Spanish-speaking population.

## 2. Materials and Methods

### 2.1. Study Design and Recruitment

This study was designed within the framework of the COVID-HL research consortium (https://covid-hl.eu, accessed on 21 February 2022), a research network on DHL related to COVID-19 in university students, which includes researchers from 50 countries including the authors of this article. This article analyzes the adaptation and validation into Spanish of the DHLI questionnaire of digital literacy applied by the COVID-HLI consortium [29]. For this study, a convenience sample was defined. Students from a public university in Extremadura-Spain, a public university in San Juan, Puerto Rico, and two universities, one public and one private, from Quito-Ecuador, were invited to participate. The invitation was made through emails addressed directly to the students as well as through the formal social networks of each of the universities. This study was conducted during the period of confinement by COVID-19 between April and June 2020. 

Data from 2318 undergraduate and postgraduate students who agreed to participate were studied: 1161 from Spain, 241 from Puerto Rico, and 917 from Ecuador. Once the students agreed to participate through providing their online informed consent, they were directed to answer the survey online through a server located in one of the participating universities.

For this study, students from all majors in the areas of Health and Life Sciences, Social Sciences, Exact Sciences, and Engineering were included.

### 2.2. Adaptation of the DHLI to Spanish

In order to carry out the cultural adaptation of the questionnaire, the original version of the article in English by Dadaczynski, Okan, and Rathmann [28] was first translated into Spanish. The translation into Spanish was carried out maintaining the semantics, conceptualization, and idiomaticity of the English version. The cultural adaptation process consisted of following the recognized methodology [32,33]: translation of the English version into Spanish by the researchers and a certified professional translator; the two versions were compared; and discrepancies were resolved. Subsequently, a reverse translation of the Spanish version into English was carried out by another translator who was not familiar with the original English version. The team that performed the first translation then compared this version with the original version, discrepancies were analyzed, and corrections were made. Words or concepts that were not clear in this version were identified, and by consensus with the authors, they were modified with words or concepts that were more common in the Spanish language. A pilot evaluation was carried out with 200 students to verify the comprehensibility of the scale. The Spanish translation was carried out by certified personnel. The adapted questionnaire, COVID-DHLI-Spanish, is described in Appendix A [34].

### 2.3. Variables and Instruments Applied

Digital Health Literacy with respect to COVID-19 (DHLI) was measured with the Spanish translated version of the Digital Health Literacy Instrument (DHLI) used by the global COVID-HL Consortium [29] and designed from the proposal of van der Vaart and Drossaert [27]. It consists of 15 items comprising five dimensions: information searching, adding self-generated content, evaluating reliability, determining relevance, and protecting privacy, each of which contains three questions. 

The methodology proposed by van der Vaart [27] was followed to create the scale. Each question has answers from one to four, where one corresponds to the worst situation and four to the best digital health literacy related to the COVID-19 situation. From this information, a categorical variable was created with the following cut-off points [30]: inadequate digital literacy (less than or equal to 2.5 points), problematic digital literacy (greater than 2.5 and less than 3 points), and sufficient digital literacy (greater than or equal to 3 points).

A survey that also contained the following variables was administered:

- Sex: Male/female/diverse;
- Age: Categorized into under 22 years old and over 22 years old;
- Country of origin: Spain, Puerto Rico, Ecuador;
- Subjective social perception: Refers to the social position of the student. This is based on the MacArthur methodology developed by Adler [35], in which the image of a staircase is established, where the tenth step is the best possible state, and the first step is the worst;
- Subjective well-being: Subjective well-being was measured with the World Health Organization (WHO) well-being scale [36]. It consists of three dimensions: sense of coherence (viewing one’s life as comprehensible, manageable, and meaningful) [37], anxiety toward the future (state of uncertainty, worry, and concern of unfavorable changes in a more remote personal future) [38,39], and well-being in the last two weeks [40]. For the creation of the subjective well-being scale, the methodology proposed by van Vogt, Jenny, and Bauer [37] for sense of coherence and the methodology of Zaleski, et al. [39] for anxiety toward the future was followed. Each question has answers from one to six or one to seven where the higher number represents better subjective well-being, and the lower one represents lower subjective well-being.

### 2.4. Statistical Analysis

Cronbach’s alpha was used for the analysis of the internal consistency of the scale and each dimension. For the validation of the content of each dimension, a principal component analysis and a varimax rotation were used to examine the theoretical fit of the five dimensions.

To measure the construct validity, the characteristics or theoretical concepts that would be related to better digital health literacy such as age, subjective social perception, sense of coherence, anxiety about the future and well-being in the last two weeks were considered. Spearman’s correlations with these variables were calculated, and a *p*-value of less than 0.05 was considered significant.

In addition, Kruskall–Wallis non-parametric mean tests were performed to observe differences in the digital health literacy scale between sociodemographic variables. Data were analyzed using Stata^®^ version 15.0 software (StataCorp LLC, College Station, TX, USA).

## 3. Results

### 3.1. Characteristics of the Participants

A total of 50.06% (*n* = 1161) of the students corresponded to universities in Spain, 39.54% (*n* = 917) to Ecuador, and 10.29% (*n* = 241) to Puerto Rico. Table 1 shows the characteristics of the participants. The majority were women (70.03%, *n* = 1624), with age less than or equal to 22 years (62.64%, *n* = 1452), coming from majors related to Life Sciences and Health (61.15%, *n* = 1418), and who were studying at the undergraduate level (88.57%, *n* = 2054). On a scale of one to 10, 26.61% (*n* = 617) of the undergraduates rated themselves as having a social status of 7. A total of 19.97% (*n* = 463) of the students reported having a chronic condition. Overall, there were no significant differences in sociodemographic characteristics between countries (Table 1).

### 3.2. Validation of the DHLI Translated into Spanish (COVID-DHLI-Spanish)

The internal consistency of the DHLI translated into Spanish (COVID-DHLI-Spanish) was good for the global scale (Cronbach’s alpha 0.69, 95% CI 0.67) as well as for each of its dimensions, except for the privacy dimension, which presented a Cronbach’s alpha of 0.45 (95% CI 0.67). Table 2 shows the results of internal consistency (Table 2).

Principal component analysis presented a chi-square with 2105 degrees of freedom of 8479.45 and *p* < 0.001, suggesting that there was sufficient correlation between each component of each dimension to be able to perform the analysis. The Kaiser Meyer Olkin sample adequacy measure was 0.86, suggesting that the sample was large enough for the analysis.

Table 3 shows the Eigenvalues of each component, with values from 1.05 to 4.81, except for the privacy protection dimension, which presented a value of less than 1. The total variance explained among all the dimensions of the scale was 65.4%. In the same table, the scores of each item after the varimax rotation are also presented; the items are grouped in the components according to the dimensions, as expected.

The DHLI scale translated into Spanish presented a direct and significant correlation with age, subjective social perception, sense of coherence, and well-being in the last two weeks; in contrast, an inverse and significant correlation was obtained with the presence of anxiety about the future (Table 4).

### 3.3. Digital Health Literacy

The mean for digital health literacy was 2.94, with a standard deviation (SD) of 0.57. The dimension that presented the highest scores was privacy protection with a mean of 3.40 ± 0.54, while the self-generated content dimension presented the lowest mean of 2.81 and SD of 0.60. According to these findings, 51.1% (*n* = 946) of the respondents were found to have sufficient digital literacy, 40.1% (*n* = 742) had problematic digital literacy, and 8.8% (*n* = 162) had inadequate digital literacy.

**Figure 1:**
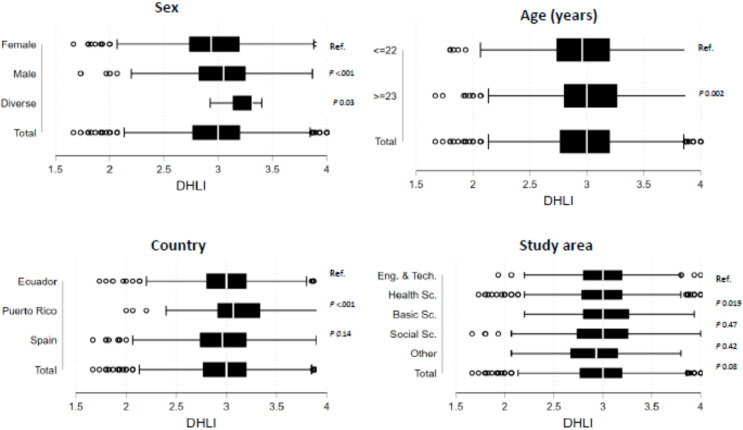
Digital health literacy scores according to the characteristics of the sample. Box plot corresponded to the score of digital literacy on COVID-19 according to the characteristics of sex, age, country of residence, study area (Eng. & Tech. = Engineering sciences and technologies; Health Sc. = Life sciences and health; Basic Sc. = Basic Science; Social Sc. = Social sciences and humanities), level of education, satisfaction with the information on COVID-19, and the presence of chronic disease in the student (No Cron. Cond = no chronic disease; Cron. Cond. = chronic disease). Next to each category were placed the *p* values obtained by the Kruskall–Wallis non-parametric test (Ref = reference).

Figure 1 presents the scores of digital literacy according to characteristics of the students. There was a significant difference between males and females, with higher digital literacy scores for males than females (3.05, SD ± 0.36 and 2.96, SD ± 0.38, respectively, *p* < 0.001). There were also significant differences in age, with those aged younger than 22 years having lower DHLI scores than those aged 22 years or older (mean 2.97, SD ± 0.36 versus mean 3.02, SD ± 0.41, *p* <0.01). Similarly, higher DHLI scores were found in those who were studying for their master’s and doctoral degrees compared to undergraduate students (mean 3.11, SD ± 0.39 and mean 2.98, SD ± 0.37, respectively, *p* < 0.001). Additionally, those who were very satisfied with COVID-19 related information found on the Internet had significantly higher DHLI scores than those who were dissatisfied (mean 3.07, SD ± 0.34 versus mean 2.95, SD ± 0.43, *p* < 0.001).

## 4. Discussion

The current COVID-19 pandemic has highlighted the importance of digital health literacy. Digital health literacy includes access to appropriate information, discrimination of messages, and care- and prevention-oriented decision making [2,9]. Health information is vital, not only for the scientific understanding of disease and its possible control measures, but also for the empowerment of citizens.

The young adult population is one of the main consumers of digital information [10], and at the same time, is at the forefront of the use of social networks for the transmission of information, which in turn, influences behaviors related to their health and care [8]. In the case of the COVID-19 pandemic, the use of digital information has become the main source of information for citizens, among which the young adult population stands out [8]. Assessing their level of digital health literacy is essential for public health actions and policies [1].

The current study analyzes the validity of the DHLI adapted to Spanish (COVID-DHLI-Spanish) to evaluate the digital health literacy related to COVID-19. The COVID-DHLI-Spanish achieved a Cronbach’s alpha of 0.69, which demonstrates a good internal reliability of the overall scale; moreover, the internal consistency was adequate for each of its dimensions, except for the privacy dimension, which obtained a Cronbach’s alpha of less than 0.5. This was similar to what was found in the validation of the original van der Vaart and Drossaert scale [27]. The reliability of the questionnaire adapted to German, Portuguese, and Korean presented a higher Cronbach’s alpha values for the global scale (0.94, 0.80, and 0.90, respectively) [16,30,31]. This difference could be explained by the fact that the authors excluded the privacy dimension in the reliability analysis as they did not consider it sensitive to evaluating DHLI [16,30,31]. In the present study, the privacy protection dimension presented an internal validity below what is desirable, which coincides with what was found in the questionnaire adapted to Portuguese and Korean, which showed limited consistency in this dimension [30,31]. This could be due to semantic problems or the lack of congruence between the questions that make it up. This finding underlines the importance of future studies that analyze the privacy protection dimension, after a review and restructuring of its components in the survey [30]. On the other hand, the results of the factor analysis suggest that there is an appropriate fit of the indicators in the five dimensions of the scale [41]. The total variance explained through all the dimensions of the scale reached 65.4%, higher than that found in the previous validation study of the DHLI into Portuguese, where the total variance reached 59.5% [30]. In this sense, it is important to mention that the digital literacy on health could be explained by other contextual elements including gender, level of education, socioeconomic perception, etc. [16,30,31] 

The DHLI scale adapted to Spanish also showed a significant correlation with age, subjective social perception, anxiety toward the future and well-being in the last two weeks, which implies adequate construct validity. This is consistent with a previous study conducted in South Korea that found appropriate construct and discriminant validity for the same questionnaire [31]. It is known that a higher age is associated with a better health literacy level [22,42,43]. On the other hand, a lower socioeconomic position is associated with a lower level of digital literacy, which has been corroborated in other studies [22,25,27,44]. Likewise, poor self-perception of health and well-being is associated with lower digital health literacy scores [22,24,40,42,45]. 

In the present study, slightly more than half of the young adult university students had sufficient digital literacy, meaning that only one in two university students had adequate digital literacy; given the context in which the study was applied, this is concerning. A total of 40.1% had problematic digital literacy, and 8.8% had inadequate digital literacy, which is similar to findings from other studies worldwide [45]. In Germany, 49.9% of university students presented adequate digital literacy in relation to COVID-19 [16]; in Pakistan, 54.3% also showed high scores, but with deficiencies in the reliability dimension [46]. In the United States, it was found that only 49% of students presented adequate digital health literacy [14]. 

Additionally, this study found higher COVID-19 related digital literacy scores in men, as reported in other studies [1]. Previous studies have remarked on women’s greater adherence to prevention and self-care practices; however, they probably encounter the most challenges in relying on available digital information [1,16]. Although gender differences in digital health literacy have not always been found [18,46], a previous study in a university population shows that men have fewer difficulties in finding information in digital media and analyzing the reliability of digital information than women [17]. 

Similarly, master’s or doctoral level students presented better digital health literacy scores, which would be explained by their better background knowledge to discern the information available in digital media and social networks, and which has been corroborated by other studies [16,46]. Likewise, those who were satisfied with the information found on the Internet had better digital health literacy scores than those who were dissatisfied with it, which agrees with other studies [46,47], suggesting that digital literacy in health would be an important predictor of satisfaction with the information. This is probably because those who are more satisfied have better background elements to access, and can interpret and use the information efficiently.

This study has some limitations, one of which is that it was not possible to carry out a concurrent validation, since there were no other scales available in its class that were adapted and validated in Spanish. Likewise, it was not possible to evaluate the validity of the scale for the different ethnic groups that inhabit the countries included in the study. Similarly, the data collection was carried out in a young adult population that regularly attended public and private universities, so there may be a bias in the information. Although it is a multicenter study, the sample was at convenience, so the population was not representative of all young university students in the three countries included. In this study, no other additional results on the associations and effects of digital literacy on health in the context of COVID-19 are presented. This analysis is part of a subsequent article.

The main advantage of this research is that it is a study that includes a population from three Spanish-speaking countries, and it is the first of its kind to analyze the validity of a scale from the digital health literacy related to COVID-19 adapted to Spanish. Previously, the same original Dadaczynski, Orkan and Rathmann [29] questionnaire has been translated into other languages including German, Portuguese, and Korean, and has consistently shown good psychometric properties [16,30,31]. This study contributes to verifying the validity of the digital literacy questionnaire related to COVID-19 in the Spanish-speaking population.

## 5. Conclusions

The digital health literacy has proven to be a key determinant to promote decision-making, behaviors, and actions in favor of health in times of pandemic. The present study aimed to analyze the validity of the DHLI scale on COVID-19 adapted to Spanish in university students and to describe its main results. The COVID-DHLI-Spanish was found to be a valid and reliable instrument to measure digital health literacy in relation to COVID-19 in Spanish-speaking populations. The authors recommend using the DHLI questionnaire adapted to Spanish to monitor and evaluate the digital health literacy in relation to COVID-19 as a valid instrument for Spanish-speaking countries.

Better digital health literacy supports sound decision-making and is associated with better health outcomes in the context of COVID-19, both individually and collectively. In this study, we observed that barely one in two university students had adequate or sufficient digital literacy on COVID-19, which is worrying, given that they are frequent users of social networks, and who, in turn, can influence the decision making by other individuals, be they colleagues, friends, family members, etc. On the other hand, this study found significant differences in digital health literacy by sex, age, and socioeconomic perception, suggesting socioeconomic and gender inequalities in relation to digital health literacy. These findings show the need for public health policies and health promotion strategies focused on strengthening the digital health literacy in the university population and in the Spanish-speaking population in general, guaranteeing equity in access to information and in the skills to manage, discriminate, and apply information to health.

## Figures and Tables

**Table 1:** Sociodemographic characteristics of participating university students from Spain, Puerto Rico, and Ecuador in 2020 (*n* = 2318).

	Total	Spain	Puerto Rico	Ecuador
Characteristic	*n* (%)	*n* (%)	*n* (%)	*n* (%)
Sex				
Female	1624 (70.03)	889 (76.57)	178 (73.86)	557 (60.74)
Male	685 (29.54)	266 (22.91)	61 (25.31)	358 (39.04)
Diverse	10 (0.43)	6 (0.52)	2 (0.43)	2 (0.22)
Age				
Under 22 years old	1452 (62.64)	700 (60.29)	136 (56.43)	617 (67.28)
Older than or equal to 22 years old	866 (37.36)	461 (39.71)	105 (43.57)	300 (32.72)
Study area				
Engineering sciences and technologies	233 (10.05)	142 (12.23)	45 (18.67)	46 (5.02)
Life sciences and health	1418 (61.15)	515 (44.36)	117 (48.55)	786 (85.71)
Basic Science	82 (3.54)	44 (3.79)	19 (7.88)	19 (2.07)
Social sciences and humanities	526 (22.68)	402 (34.63)	59 (24.48)	65 (7.09)
Another	60 (2.59)	58 (5)	1 (0.41)	1 (0.11)
Level of education				
Undergraduate	2054 (88.57)	1042 (89.75)	144 (59.75)	868 (94.66)
Master	178 (7.68)	86 (7.41)	68 (28.22)	24 (2.62)
Other (e.g., Ph.D.)	87 (3.75)	33 (2.84)	29 (12.03)	25 (2.73)
Subjective social status (10 steps)				
1	3 (0.13)	1 (0.09)	1 (0.41)	1 (0.11)
2	22 (0.95)	8 (0.69)	4 (1.66)	10 (1.09)
3	111 (4.79)	40 (3.45)	20 (8.3)	51 (5.56)
4	200 (8.62)	86 (7.41)	28 (11.62)	86 (9.38)
5	471 (20.31)	167 (14.38)	60 (24.9)	244 (26.61)
6	539 (23.24)	283 (24.38)	60 (24.9)	196 (21.37)
7	617 (26.61)	375 (32.3)	39 (16.18)	203 (22.14)
8	304 (13.11)	171 (14.73)	20 (8.3)	113 (12.32)
9	34 (1.47)	21 (1.81)	6 (2.49)	7 (0.76)
10	18 (0.78)	9 (0.78)	3 (1.24)	6 (0.65)
Chronic condition				
No	1856 (80.03)	923 (79.5)	182 (75.52)	751 (81.9)
Yes	463 (19.97)	238 (20.5)	59 (24.48)	166 (18.1)

**Table 2:** Internal consistency of the DHLI questionnaire translated into Spanish, COVID-DHLI-Spanish (*n* = 2318).

	Cronbach’s Alpha	Unilateral CI (95%)
Global	0.69	0.67
Dimensions Information searching	0.79	0.78
Self-generated content	0.76	0.74
Reliability	0.74	0.72
Relevance	0.73	0.71
Privacy protection	0.45	0.41

**Table 3:** Principal component analysis of the dimensions of the COVID-DHLI-Spanish ^a^.

	Component				
Item	1	2	3	4	5
Information searching dimension					
When you search the Internet for information on coronavirus or related topics, how easy or difficult is it for you to…					
1. Make a choice from all the information you find?	0.54				
2. Use the proper words or search query to find the information you are looking for?	0.56				
3. Find the exact information you are looking for?	0.54				
Self-generated content dimension					
When typing a message (on a forum, social network) about coronavirus or related topics, how easy or difficult is it for you to…					
4. Clearly formulate your question or health-related worry?			0.48		
5. Express your opinion, thoughts, or feelings in writing?			0.62		
6. Write your message as such, for people to understand exactly what you mean?			0.59		
Reliability dimension					
When you search the Internet for information on coronavirus or related topics, how easy or difficult is it for you to…					
7. Decide whether the information is reliable or not?				0.49	
8. Decide whether the information is written with commercial interests (e.g., advertising, trying to sell products)?				0.57	
9. Check different websites to see whether they provide the same information?				0.37	
Relevance dimension					
When you search the Internet for information on coronavirus or related topics, how easy or difficult is it for you to…					
10. Decide if the information you find is applicable to you?		0.35			
11. Apply the information you found in your daily life?		0.56			
12. Use the information you found to make decisions about your health (e.g., protective measures, hygiene regulations, transmission routes, risks, and their prevention?		0.58			
Privacy protection dimension					
When you post a message (on a forum, social network) about coronavirus or similar topics, how often…					
13. Do you find it difficult to judge who can read along?					0.28
14. Do you share (intentionally or unintentionally) your own private information (e.g., name or address)?					0.66
15. Do you share (intentionally or unintentionally) someone else’s private information?					0.67
Eigenvalue	4.81	1.58	1.48	1.05	0.90
Percentage of variance	15.84	13.57	13.51	12.62	9.89

^a^ The scale and the dimensions of the scale with their respective questions correspond to the English version of the original DHLI questionnaire designed by Dadaczynski, Okan, and Rathmann [9].

**Table 4:** Construct validity of the DHLI scale translated into Spanish with the Spearman correlation.

Variable	Rho (ρ)	*p*-Value
Age	0.09	<0.001
Subjective social perception	0.09	<0.001
Sense of coherence	0.18	<0.001
Anxiety about the future	−0.23	<0.001
Well-being on the last two weeks	0.18	<0.001

## Data Availability

The data presented in this study are available on request from the corresponding author.

## Appendix A

Spanish translated version of the Digital Health Literacy Instrument (DHLI) [12], based on the English version by Dadaczynski, Okan, and Rathmann [9].

ENCUESTA COVID-HL: ESTUDIANTES UNIVERSITARIOS

Empezamos preguntándote alguna información personal

1. Por favor, indica tu sexo

- Femenino
- Masculino
- Diverso

2. ¿Qué edad tienes?

Tengo _______ años

Ahora las preguntas hacen referencia a cómo de fácil o difícil es para ti tratar la información sobre el coronavirus.

3. ¿Has buscado información sobre el coronavirus en internet en las últimas 4 semanas? Esto puede incluir, por ejemplo, información sobre casos infectados, cómo evitar o gestionar las restricciones en la vida cotidiana.

- Sí, solo información para mí.
- Sí, solo información para otras personas.
- Sí, información para mí y para otras personas.
- No, no he buscado información en las últimas 4 semanas

4. Cuando buscas información en internet sobre el coronavirus o temas relacionados, ¿Cómo de fácil o de difícil es para ti en relación a lo siguiente:…

Opciones: Muy fácil, Fácil, Difícil, Muy difícil

- Seleccionar entre toda la información que has encontrado?
- Utilizar las palabras correctas para encontrar aquello que buscas o para consultar sobre lo que buscas?
- Encontrar la información exacta que estás buscando?

5. Cuando escribes un mensaje (en un foro, red social) sobre el coronavirus o temas relacionados, ¿Cómo de fácil o difícil es para ti:…

Opciones: Muy fácil, Fácil, Difícil, Muy difícil

- Formular claramente tu pregunta o tu preocupación relacionada con la salud?
- Expresar tu opinión, pensamientos, o sentimientos por escrito?
- Escribir tu mensaje de forma que la gente entienda lo que quieres decir exactamente?

6. Cuando buscas información en internet sobre el coronavirus o temas parecidos, ¿Cómo de fácil o difícil es para ti…

Opciones: Muy fácil, Fácil, Difícil, Muy difícil

- Determinar si la información es fiable o no?
- Diferenciar si la información tiene relación con intereses comerciales (Por ej.: publicidad, intentando vender productos)?
- Revisar diferentes páginas web para ver si proporcionan la misma información?

7. Cuando buscas información en internet sobre el coronavirus o temas parecidos, ¿Cómo de fácil o difícil es para ti…

Opciones: Muy fácil, Fácil, Difícil, Muy difícil

- Decidir si la información que encuentras es útil en tu caso?
- Aplicar la información encontrada en tu vida diaria?
- Utilizar la información encontrada para tomar decisiones sobre tu salud (Por ej.: medidas de protección, normativas de higiene, vías de transmisión, riesgos y su prevención?

8. Cuando publicas un mensaje (en un foro, red social) sobre el coronavirus o temas parecidos, ¿Con qué frecuencia…

Opciones: Nunca, Una vez, Varias veces, Muchas veces

- Encuentras difícil averiguar quién lo puede leer?
- Compartes (intencionalmente o sin querer) tu información personal (Por ej.: nombre o dirección)?
- Compartes (intencionalmente o sin querer) la información privada de otra persona?

A continuación, se aborda la situación actual de la propagación del coronavirus en tu país y en todo el mundo. Las siguientes preguntas se refieren a la búsqueda de información relacionada con el coronavirus y las consecuencias para tu vida personal.

9. En este momento hay varias posibilidades de obtener información sobre el coronavirus y temas relacionados en internet. Por favor, indica con qué frecuencia usas en la actualidad las siguientes fuentes

Opciones: Frecuentemente, A veces, Casi nunca, Nunca, No sé

- Navegadores (Por ej.: Google, Bing, Yahoo u otro)
- Páginas web de cuerpos públicos (Por ej.: Ministerio de Salud, Ministerio de Educación, Policía Nacional)
- Wikipedia u otras enciclopedias virtuales
- Redes sociales (Por ej.: Facebook, Instagram, Twitter)
- YouTube
- Blogs de temas de Salud
- Guías de comunidades (Por ej.: De pacientes, grupos religiosos, activistas, sindicatos)
- Páginas web de médicos o compañías de salud privadas.
- Nuevas Web (Por ej: de Periódicos y Canales de Televisión).

10. ¿En qué idioma están las fuentes que consultas sobre coronavirus y temas relacionados?

Puedes seleccionar varias opciones de respuesta si fuera necesario.

- Español
- Inglés
- Francés
- Portugués
- Lenguas cooficiales en España (Catalán, Euskera, Gallego, Aranés)
- Otro. Especificar…….

11. Por favor, indica los temas concretos que has buscado en relación al coronavirus. Puedes seleccionar varias opciones de respuesta si fuera necesario.

- Actual propagación del coronavirus (Por ej.: número de casos infectados)
- Vías de transmisión del coronavirus.
- Síntomas de la enfermedad por COVID-19.
- Medidas de protección individuales frente a la infección (Por ej.: consejos para lavarse las manos, etc).
- Normas de higiene (Por ej.: desinfección y limpieza)
- Evaluaciones e informes de la situación actual y recomendaciones (Por ej.: del Ministerio de Sanidad, Consumo y Bienestar Social; Otras Administraciones públicas)
- Restricciones (Por ej.: Restricciones de salidas, y órdenes de confinamiento)
- Consecuencias económicas y sociales del coronavirus.
- Relacionado con el estrés psicológico causado por el coronavirus.
- Otro. Especificar………

12. Ahora se trata de conocer lo que valoras cuando buscas información en internet sobre el coronavirus o temas relacionados.

- ¿Cómo de importante es para ti que:

Opciones: Muy importante—Bastante importante—Poco importante—Nada importante

- La información esté actualizada?
- La información haya sido confirmada?
- Enterarte rápidamente de las cosas más importantes?
- La información venga de fuentes oficiales?
- Represente las diferentes opiniones?
- El tema se haya tratado integralmente?

13. ¿Cuál es tu grado de satisfacción con la información encontrada en internet sobre el coronavirus?

- Muy insatisfecho
- Insatisfecho
- Parcialmente satisfecho
- Satisfecho
- Muy satisfecho
